# NKG2D: A Master Regulator of Immune Cell Responsiveness

**DOI:** 10.3389/fimmu.2018.00441

**Published:** 2018-03-08

**Authors:** Felix M. Wensveen, Vedrana Jelenčić, Bojan Polić

**Affiliations:** ^1^Department of Histology and Embryology, Faculty of Medicine, University of Rijeka, Rijeka, Croatia

**Keywords:** NKG2D, education, peripheral tolerance, activation, NK cells, T cells

## Abstract

NKG2D is an activating receptor that is mostly expressed on cells of the cytotoxic arm of the immune system. Ligands of NKG2D are normally of low abundance, but can be induced in virtually any cell in response to stressors, such as infection and oncogenic transformation. Engagement of NKG2D stimulates the production of cytokines and cytotoxic molecules and traditionally this receptor is, therefore, viewed as a molecule that mediates direct responses against cellular threats. However, accumulating evidence indicates that this classical view is too narrow. During NK cell development, engagement of NKG2D has a long-term impact on the expression of NK cell receptors and their responsiveness to extracellular cues, suggesting a role in NK cell education. Upon chronic NKG2D engagement, both NK and T cells show reduced responsiveness of a number of activating receptors, demonstrating a role of NKG2D in induction of peripheral tolerance. The image that emerges is that NKG2D can mediate both inhibitory and activating signals, which depends on the intensity and duration of ligand engagement. In this review, we provide an overview of the impact of NKG2D stimulation during hematopoietic development and during acute and chronic stimulation in the periphery on responsiveness of other receptors than NKG2D. We propose that NKG2D interprets the context of the immunological environment through detection of cellular cues and in response sets the appropriate activation threshold for a large number of immune receptors. This perspective is of particular importance for future therapies that aim to exploit NKG2D signaling to fight tumors or infection.

## Introduction

NKG2D, encoded by *Klrk1*, is an activating cell surface receptor that is predominantly expressed on cytotoxic immune cells. NKG2D is abundantly present on all NK cells, NKT cells, and subsets of γδ T cells. While naïve human CD8^+^ T cells express NKG2D, in mice they upregulate its expression only after activation (1). CD4^+^ T cells generally do not express NKG2D even after activation, but in humans its expression can be induced under certain pathological conditions, such as Crohn’s disease juvenile-onset lupus and cytomegalovirus infection (2–4). In mice, CD4 T cells were shown to induce NKG2D expression in models for inflammation, such as colitis and chronic inflammatory arthritis (5, 6). The molecular structure of NKG2D allows it to bind a number of structurally different MHC-I-like ligands. NKG2D ligands have in common that under homeostatic conditions their expression is generally low. In contrast, upon cellular stress, such as infection or oncogenic transformation, their expression can be highly induced (7). In humans, the NKG2D ligands are MICA, MICB, and six members of the ULBP family. In mice, ligands can be divided into three subgroups: five different isoforms of the Rae1-family (α-ε), MULT1, and three different isoforms of H60 (a, b, and c) (7).

The NKG2D receptor consists of a homodimer of two disulfide-linked transmembrane proteins, with very short intracellular domains that do not have signaling properties. In mice, NKG2D therefore uses the adaptor molecules DAP10 and DAP12 to relay its signaling, whereas in humans NKG2D associates exclusively with DAP10 (8). Two NKG2D isoforms have been identified in mice, a short (NKG2D-S) and a long (NKG2D-L) form, which differ 13 amino acids in length as result of alternative splicing of the *Klrk1* transcript (9). Due to this difference in length, NKG2D-L can only associate with DAP10, whereas NKG2D-S can form a complex with both DAP10 and DAP12. In humans, only the NKG2D-L isoform is expressed explaining why this receptor exclusively signals through DAP10 (10, 11). DAP10 and DAP12 initiate different signaling cascades. DAP10 possesses a YINM motif which allows binding p85 of phosphatidylinositol-3 kinase (PI3K) (12). In addition, DAP10 binds Grb2, which associates with Vav1. All three of these molecules are required to mediate the full signaling potential of NKG2D over DAP10 (13). DAP12 contains an immune receptor tyrosine-based activation motif, which is phosphorylated by Src-kinases upon NKG2D triggering (14). This event allows binding and activation of the tyrosine kinases, Syk and Zap70 (12). T cells and naïve NK cells predominantly express the NKG2D-L isoform, which is therefore thought to promote cellular processes downstream of the PI3K signaling cascade, such as co-stimulation, cytotoxicity, and cell survival (15–17). In mice, NKG2D-S is induced in activated NK cells, in which it promotes signaling through Syk/Zap70, resulting in enhanced cytotoxicity and cytokine production (17).

NKG2D plays an important role in the recognition and elimination of potentially dangerous cells (1, 18). It has been shown to mediate immune responses against tumors (18), virally infected cells (8, 19), and organ transplants (20). For this reason, NKG2D was originally thought to predominantly mediate direct cytotoxicity in response to the encounter of ligand on stressed target cells (1). However, in most cases, NKG2D is only able to mediate immune cell activation if it occurs within an inflammatory context. Both NK and T cells generally require a secondary signal before NKG2D is able to mediate a measurable effect (21–23). The primary function of NKG2D therefore appears to be regulation of signaling through other receptors. Its unique feature is that it is able to both inhibit and potentiate signaling of a large number of receptors in multiple ontologically distinct immune cell subsets and during different stages of the life cycle of immune cells, such as hematopoietic development, priming, and effector responses (8). In this review, we will give a brief overview of the literature regarding the role of NKG2D in various immunological settings. The model that emerges from accumulated evidence is that NKG2D is a master regulator of activation thresholds for a large number of receptors, both when NKG2D is directly engaged, and long after its signaling has ceased.

## NKG2D and NK Cells

As part of innate immunity, NK cells play an important role in the early cytolytic defense against infections and tumors. NK cells are members of the type 1 family of innate lymphoid cells (24, 25). On their cell surface they express a large number of structurally distinct, germline-encoded receptors that can transfer both activating and inhibitory signals into the cell (26). These receptors respond to external cues from peripheral cells that communicate either inhibitory homeostatic signals, or activating signals in case of cellular stress (7). Under normal conditions inhibitory signals prevail, which keeps NK cells inactive. When cells become stressed, for example, upon infection or oncogenic transformation, activating signals dominate causing loss of equilibrium and NK cell activation (27). To prevent autoimmunity or anergy, an extensive set of regulatory mechanisms is in place that determines the activation threshold values beyond which the balance between inhibitory and stimulating cues shifts in favor of activation. Already during their development, in a process known as “education,” “licensing,” or “arming,” NK cell activation thresholds are set, mostly in response to inhibitory receptors. NK cell education ensures proper reactivity, as well as tolerance toward self in response to locally expressed ligands. This process functionally mimics positive and negative selection of T cells in the thymus (28–30). Outside of the bone marrow, the responsiveness of NK cells is further fine-tuned by engagement of self-ligands by their receptor array, which mediates peripheral tolerance.

**Figure 1:**
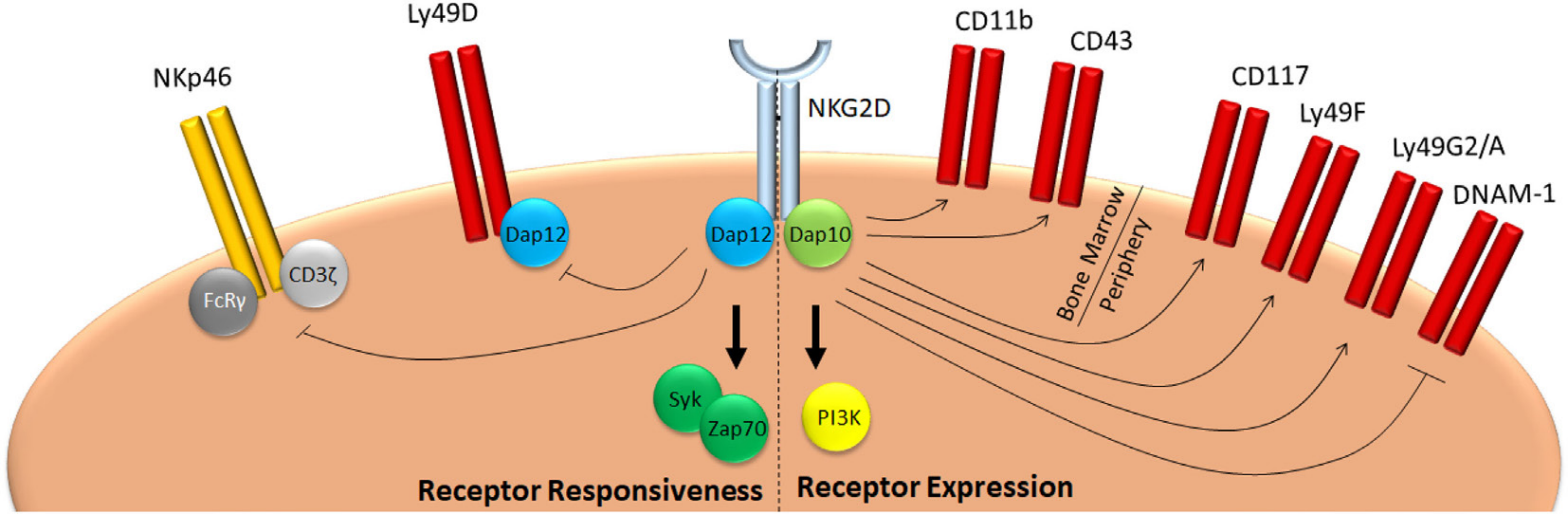
NKG2D mediates education of NK cells. NKG2D signaling during development negatively affects responsiveness of NK cells in two ways. On one hand, it promotes expression of inhibitory Ly49 molecules, such as Ly49F, G2, or A, whereas it inhibits expression of activating receptors, such as DNAM-1. On the other, it reduces responsiveness of activating receptors, such as NKp46 and Ly49D. As a consequence, mice deficient for NKG2D have a hyper-responsive phenotype and are better protected against infection with pathogens which are controlled by NK cells, such as cytomegalovirus (33, 34).

NKG2D is expressed from the earliest NK cell precursor stages onward (31). Initially, its expression is relatively low, but increases over time and stays high in mature cells (32). In mice, NK cells express both NKG2D isoforms, even though the long form predominates in a resting state (9). Expression of the NKG2D-S isoform strongly increases after NK cell activation, whereas levels of the L-form abate (10). Nevertheless, both DAP10 and DAP12-mediated signaling is engaged upon NKG2D stimulation in activated murine NK cells (15). NKG2D has been implied in NK cell education, effector cell function, and peripheral tolerance through modification of the activation threshold of NK cell receptors. During development, NKG2D regulates both expression levels and responsiveness of a plethora of receptors (Figure 1). NK cells of mice with a germline deficiency for NKG2D show reduced levels of c-kit (CD117), the activating receptor DNAM-1, as well as the inhibitory receptors Ly49A, Ly49G2, and Ly49F (33, 34). In addition, whereas *Klrk1^−/−^* NK cells fail to respond to target cells expressing NKG2D ligands (33, 35), mice lacking either NKG2D, or its ligands Rae1δ and Rae1ε produce higher levels of IFNγ following stimulation with cellular targets (33, 34, 36). As a result, NKG2D-deficient mice display better NK cell-mediated control of murine cytomegalovirus infection (33). This effect seems specific for NKG2D, since deletion of CD16, another activating receptor expressed on all NK cells, does not affect CD16 independent NK cell function both in humans and in mice (37–39).

Humans do not express Ly49 molecules, nor does human NKG2D engage DAP12. Unfortunately, the impact of NKG2D on NK cell development in humans is difficult to determine. To date, no deficiency for this receptor has been documented and in the periphery most NK cells express NKG2D, precluding comparison of NK cell subpopulations with and without this receptor. This makes it difficult to translate many observations made in NKG2D-deficient mice directly to the human situation. However, the maternal decidua contains a large population of NKG2D^−^ NK cells, which is replaced by NKG2D^+^ cells during the first trimester of pregnancy (40). Notably, this is associated with strong changes in the KIR repertoire and reduced pro-inflammatory NK cell function (40, 41). These observations suggest that also in humans NKG2D may affect NK cell development and education.

The impact of NKG2D on NK cell development is likely to depend on its interaction with the IL-15 receptor (IL-15R). IL-15R signaling is known to be important for the development, homeostasis, and survival of NK cells (42). Both NKG2D and the IL-15R were shown to be able to bind DAP10 in murine cells (16). Jak3, as part of the canonical IL-15R signaling pathway, phosphorylates the YINM sequence on DAP10 and was shown to be able to potentiate NKG2D signaling (16). *Klrk1*^−^*^/^*^−^ mice show a higher proliferation rate as well as faster differentiation and transition to more mature stages resulting in perturbation in size of immature NK cell subpopulations (33). Importantly, NKG2D-deficient NK cells are prone to apoptosis, which could not be corrected by addition of IL-15 (33), suggesting a common signaling pathway. Both NKG2D and the IL-15R can activate PI3 kinase, a molecule important for the regulation of proliferation and survival (43). It therefore seems likely that the PI3K pathway is engaged by these receptors to mediate common regulatory effects. Whether NKG2D prevents a general hyper-reactivity of NK cells or if it sets the activation threshold of a specific activating NK cell receptor still needs to be investigated.

**Figure 2:**
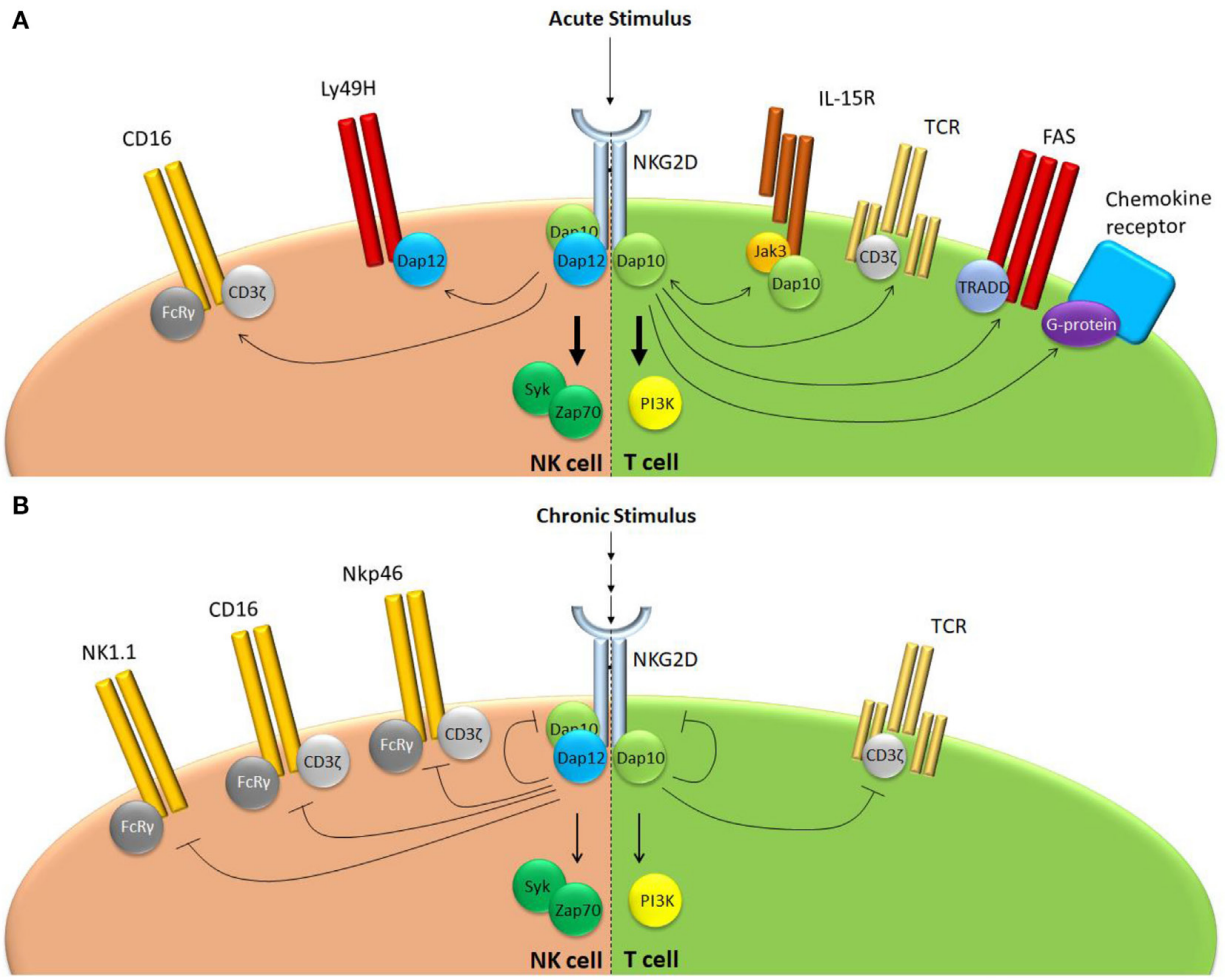
NKG2D regulates receptor responsiveness differently following acute or chronic stimulation. **(A)** Following acute stimulation, NKG2D promotes responsiveness of a range of structurally unrelated receptors that use largely distinct intracellular signaling modalities. **(B)** Chronic NKG2D engagement mediates its own downregulation and subsequent hypo-responsiveness to stimulation. In addition, chronic NKG2D stimulation impairs T cell receptor responsiveness in T cells. In NK cells, chronic NKG2D stimulation reduces missing self-signaling (not shown) as well as responsiveness of a number of receptors that share the FcεRIγ signal adaptor.

NKG2D plays a key role in effector responses of NK cells in the periphery (Figure 2). NKG2D itself is an important mediator of tumor immuno-surveillance, since animals deficient for NKG2D demonstrate a reduced ability to fight prostate carcinoma and B cell lymphoma, but not chemically induced fibrosarcoma (35). In addition, NKG2D ligation was shown to reduce activation thresholds for several NK receptors, both in humans and in mice. In human NK cells, NKG2D promotes CD16 signaling and ADCC, since blocking of NKG2D receptors results in a reduced ability of NK cells to mediate anti-HIV-1 antibody-dependent cellular cytotoxicity (44). Conversely, NKG2D was shown to be able to synergistically activate human NK cells when they were simultaneously stimulated through CD16, NKp46, or 2B4 (23, 45). For the 2B4-NKG2D pair, it was shown that synergy is achieved through conversion on the signaling adaptor Vav1, which overcomes c-Cbl-mediated inhibition (46). In mice, NKG2D was shown to augment Ly49H-dependent proliferation (47). Wild type Ly49H^+^ NK cells proliferate faster than their *Klrk1*^−^*^/^*^−^ counterparts following infection with a virus that drives overexpression of the NKG2D ligand Rae1γ (47). Both NKG2D and Ly49H use DAP12 for their signal transduction providing a possible overlap in signaling cascades. It should be noted, however, that the Rae1γ transgenic virus mediates important NKG2D-independent effects (48) and additional evidence is required before NKG2D and Ly49H signaling can be directly linked.

NKG2D has also been implied in the generation of peripheral tolerance of NK cells, an effect that was first identified in cancer patients. Whereas some tumors downregulate NKG2D ligands to prevent recognition, others paradoxically induce expression NKG2D ligands (49). Rather than activating NK cells, high NKG2D ligand expression mediates downregulation of the NKG2D receptor on tumor infiltrating lymphocytes (TILs) and reduced responsiveness to NKG2D engagement (50). Under physiological conditions, this process is thought to prevent NK hyper-responsiveness against peripheral ligands for which education in the bone marrow is incomplete. In addition, it allows induction of NK cell hypo-responsiveness at times when this is required, for example during pregnancy (41). The human placenta was shown to produce both soluble and exosome-bound NKG2D ligands. During pregnancy, the placenta accumulates a large number of NK cells, which are inhibited from recognizing the fetus as non-self. Both supernatants of umbilical cord cells and placental exosomes were shown to downmodulate NKG2D and inhibit NK cell cytotoxicity (51–53). Loss of NK cell functionality after exosome treatment could be prevented using blocking antibodies directed against NKG2D. NKG2D-induced hypo-responsiveness was therefore proposed as a mechanism to mediate fetal tolerance. Indeed, several polymorphisms in NKG2D and MICA are associated with recurrent miscarriage (54).

Chronic NKG2D engagement by NK cells results in reduced responsiveness of multiple receptors other than NKG2D. An impaired ability to kill RMA-S cells was observed in mice that transgenically overexpress Rae1ε or MICA and upon chronic exposure of NK cells to NKG2D ligands *in vitro*. This indicates a role for NKG2D in regulation of missing self-recognition (55–57). When murine NK cells were long-term exposed to cells overexpressing the NKG2D ligand H60, they showed reduced responsiveness to CD16, NK1.1, and NKp46 stimulation (55). NKG2D-induced peripheral tolerance demonstrated receptor specificity, since Ly49H and Ly49D function were reduced, but still partially preserved (55). Similarly, when human NK cells were chronically exposed to MICA, downregulation of CD3ζ was observed, rendering them hypo-responsive to CD16, NKp46, and NKp30, but not 2B4 stimulation (58). In mice, NK cell hypo-responsiveness following chronic NKG2D engagement requires signaling through both DAP10 and DAP12 adaptor molecules (55). Interestingly, most receptors affected by chronic NKG2D exposure, such as NK1.1, NKp46, or CD16, do not make complexes with either DAP10 or DAP12, but rather use the adaptor molecule FcεRIγ (59). This implies that prolonged NKG2D stimulation influences signaling components downstream of FcεRIγ. Again, it seems that this level of regulation is specific for NKG2D since sustained exposure of murine NK cells to CD16, another broadly expressed NK cell activating receptor, did not affect any other activation pathway besides CD16-mediated NK cell activation (55). NKG2D-mediated peripheral tolerance appears to depend on the level of stimulation. Chronic exposure of murine NK cells to Rae1ε or Rae1δ, NKG2D ligands of relatively low affinity, induced general hypo-responsiveness of NK cells to activating stimuli (“desensitization”). Tolerance could be broken through administration of soluble MULT1, an NKG2D ligand that binds this receptor with high affinity (36). How this effect is mediated molecularly, or why a difference in the affinity of NKG2D ligands has such opposing effects on NK cell activity is still unknown.

In summary, NKG2D affects all stages of the life cycle of NK cells through modification of NK cell receptor activation thresholds. In addition to its important role in recognition and elimination of potentially dangerous cells, NKG2D mediates NK cell education in the bone marrow and peripheral tolerance upon chronic ligand exposure. Further investigation is needed to determine how NKG2D affects activity of receptors that do not share its downstream signaling components.

## NKG2D as a Modulator of Receptor Responsiveness in T Cells

Inappropriate activation of T cells, for example by self antigens, may lead to devastating tissue destruction. Therefore, priming of naïve T cells requires not only T cell receptor (TCR) engagement, but also co-stimulation via membrane-bound receptors and/or cytokines (60). Co-stimulatory molecules ensure survival of T cells and control responsiveness of TCR engagement of effector cells in the periphery. The most well-documented co-stimulatory molecule is CD28. Upon engagement of B7 molecules, this protein activates several signaling cascades, including the NF-kB and PI3 kinase pathways (61). Deficiency of CD28 causes a reduction in proliferation and cytokine production by T cells upon infection (61). T cells expressing a mutant form of CD28 that lacks the PI3K binding YMNM motif have a specific impairment in producing cytokines (62). This indicates that co-stimulatory functions are associated with specific signaling cascades. Indeed, deficiency of CD28 does not abrogate the cytotoxic response against LCMV infection (63), demonstrating that co-stimulation does not necessarily induce a general state of increased responsiveness, but is able to potentiate specific receptors.

NKG2D has been well-documented as a co-stimulatory molecule for T cells. Upon MICA engagement, activated human CD8 T cells produce more IFNγ, TNF, and IL-2 in response to TCR stimulation (22). Moreover, the cytolytic response of antigen-specific CD8 T cells against peptide-pulsed target cells is enhanced if these cells overexpress ligands for NKG2D (22, 64). For priming of naïve CD8 T cells, NKG2D is insufficient as a co-stimulatory molecule. *In vitro* stimulation of human peripheral blood CD8 T cells showed that CD28-mediated co-stimulation is required for NKG2D to be able to promote cytokine production and cytotoxicity following TCR stimulation (65). The *in vivo* role of NKG2D on T cells long remained elusive, because NKG2D deficiency on NK cells results in their hyper-responsiveness, thus affecting viral titers and the subsequent CD8 T cell responses (33, 66). Specific elimination of NKG2D on CD8 T cells demonstrated that this receptor has a non-redundant role in promoting cytokine production following viral infection in mice (66).In contrast, deficiency of NKG2D on these cells did not impact their ability to expand or mediate cytotoxicity (66). Conversely, when mice are infected with a cytomegalovirus strain that transgenically expresses the NKG2D ligand Rae1γ, no major differences in effector CD8 T cell numbers are observed, but these cells do display increased cytokine production and cytotoxic potential (48). This indicates that NKG2D is able to promote cytotoxicity in antiviral CD8 T cells, but is not required by these cells to obtain their normal cytolytic potential.

In contrast to αβ T cells, γδ T cells do not require priming and are directly able to mediate cytokine production and cytolytic killing upon TCR engagement (67). γδ T cells constitutively express NKG2D. However, most γδ T cells are not able to mediate cytotoxicity upon NKG2D triggering alone (68, 69). Indeed, also in γδ T cells does NKG2D function to potentiate TCR responsiveness with regards to cytokine production and target cell lysis (68, 70).

The TCR functionally defines the T cell and, therefore, has been given most attention regarding its interaction with NKG2D. However, NKG2D mediates sensitization of receptors in T cells beyond the TCR (Figure 2). Most notably, NKG2D has been associated with IL-15 receptor signaling and memory CD8 T cell formation. Upon stimulation, the IL-15 receptor activates the adaptor molecules Jak1 and Jak3, which mediate phosphorylation of Stat5, but also DAP10, thus potentiating NKG2D signaling through DAP10 (16). Indeed, both DAP10 expression and IL-15 stimulation mediate NKG2D expression on the surface of murine effector CD8 T cells (15, 71). Conversely, NKG2D potentiates IL-15R signaling in memory CD8 T cell precursors. Upon LCMV infection, murine CD8 T cells deficient for NKG2D have a reduced capacity to form central memory cells, which is DAP10 and IL-15 dependent (72). In absence of NKG2D, memory precursor CD8 T cells show reduced phosphorylation of Akt, a downstream target of PI3 kinase, following IL-15 stimulation. As a result, these cells have a reduced ability to sustain levels of the pro-survival protein Mcl-1, leading to lower number of central memory cells (72). Thus, both in NK and CD8 T cells, NKG2D and the IL-15R potentiate each other’s signaling through DAP10 even though the functional outcome differs between these cell subsets. In murine memory T cell precursors, NKG2D also mediates downregulation of the transcription factor T-bet by limiting JNK2 signaling (73). Whether this effect is downstream of the IL-15R or of another ligand was not shown (73).

FAS, also known as CD95, is a member of the TNF receptor superfamily. Dependent on the intracellular adaptor molecules that are associated to this receptor, FAS ligation may induce apoptosis, growth arrest, or proliferation (74). In a large number of human tumors, infiltrating CD4 T cells were shown to express high levels of NKG2D (75). Rather than inducing a cytolytic response, MICA expression by these tumors induced expression of FASL by NKG2D-expressing CD4 T cells. Whereas, tumor cells themselves were protected against FASL-induced apoptosis, it did mediate growth arrest in NKG2D^−^, but not in NKG2D^+^ cells (75). In glioblastoma cells, FAS was shown to recruit the p85 subunit of PI3 kinase, which enhanced invasive growth (76). Since NKG2D/DAP10 signals through PI3K in T cells, it seems likely that this mechanism is adopted in tumor infiltrating T cells to prevent FASL-mediated growth arrest.

Finally, NKG2D stimulation affects responsiveness of T cells to chemokines. Deficiency of NKG2D does not result in changes in migration *per se*, as naïve or effector T cells of NKG2D-deficient mice do not show differences in tissue homing (66). However, in a mouse that transgenically overexpressed the NKG2D ligand Rae1ε in pancreatic β-islet cells, CD8 T cells rapidly accumulated in the pancreas, which was associated with strong changes in the local chemokine milieu (77). This effect was dependent on DAP10, but did not require antigen-recognition by T cells (77). Surprisingly, accumulation of CD8 T cells in the pancreas of these animals did not lead to massive destruction of β-cells or diabetes. The molecular mechanism how NKG2D impacts chemotaxis is not known, but the involvement of DAP10 again implies a role for PI3 kinase. Many chemokine receptors signal through PI3 kinase, either directly, or via G-protein linked signaling (78). Thus, NKG2D may indirectly impact the sensitivity of PI3K-linked chemokine receptors to change the migration behavior of T cells.

Similar to NK cells, chronic exposure of T cells to NKG2D ligands impairs their responsiveness to stimulation. Characterization of T cells infiltrating human tumors overexpressing NKG2D ligands showed that they downregulate the NKG2D receptor (50). As a result, activated T cells become less responsive to NKG2D (co-) stimulation (50). Surprisingly, chronic NKG2D ligand encounter also results in reduced responsiveness of the TCR. Re-stimulation of TILs from a MICA overexpressing human melanoma through their TCR showed that they have a reduced capacity to produce IFNγ compared to TILs derived from melanoma that did not express NKG2D ligands (50). To recapitulate this observation, various experimental models have been generated in which mice overexpress NKG2D ligands, either ubiquitously or in specific organs. These confirmed that chronic NKG2D stimulation impairs responsiveness to tumor antigens in particular (57, 79). The molecular mechanism underlying impaired responsiveness of the TCR following chronic NKG2D stimulation is still a matter of debate. Co-cultivation of human peripheral blood CD8 T cells for several days with fibroblasts overexpressing MICA showed that these cells downregulated CD3ζ, a key intracellular signaling component of the TCR (58). This effect was shown to depend on activation of caspase 3/7, which mediated proteolytic degradation of CD3ζ (58). How chronic NKG2D drives caspase activation and how this is achieved without inducing apoptosis remains unclear. Interestingly, when animals which ubiquitously express Rae1ε were infected with murine cytomegalovirus, there was no difference observed in the number of antigen-specific CD8 T cells, nor in their ability to produce cytokines (80). This implies that anergy induced by chronic NKG2D stimulation can be overcome in a sufficiently inflammatory environment (80).

## NKG2D and B Cells

A surprising role for NKG2D was recently identified in regulation of signaling thresholds in B cells. Mice deficient for NKG2D show a specific reduction in the number of B cells in spleen (33). In addition, *Klrk1*^−^*^/^*^−^ mice have a twofold reduction in B1a cells in the peritoneal cavities (81). This phenotype partially depended on DAP10-mediated signaling, indicating that this may also be of relevance for the human situation. NKG2D is not expressed on mature B cells or committed B cell precursors. Nevertheless, NKG2D is expressed very early during NK cell development. Since B1a cells represent an innate B cell population, it was postulated that in mice NKG2D is expressed on a common precursor for B1a and NK cells (81). Importantly, in a model for B1a cell controlled bacterial infection, *Klrk1*^−^*^/^*^−^ mice behaved similarly to B cell-deficient animals (81). Indeed, NKG2D deficiency resulted in reduced B cell receptor signaling in B1a cells. Thus, also in the B cell lineage, NKG2D appears to have a long-term impact on receptor sensitivity.

## Concluding Remarks

NKG2D has great potential as a therapeutic target, since it has the potency to enhance cytolytic immune responses against important diseases, such as cancer. Indeed, chimeric receptors using NKG2D signaling domains have successfully been used *in vitro* to potentiate antitumor T cells (82, 83). However, the complexity of NKG2D biology should be acknowledged before the power of this receptor can be harnessed therapeutically. NKG2D ligands may be used to communicate cellular stress, in which case NKG2D should induce immune cell activation. However, when NKG2D ligands are expressed chronically or at low intensity, for example during pregnancy in the placenta, they communicate a need for reduced receptor responsiveness and inhibit immune cell function. Similarly, when NKG2D ligands are encountered by NK cell precursors they set the proper bandwidth for mature NK cell responsiveness. Thus, NKG2D appears to be a sensor of the immunological context in which an immune cell operates and it adjusts responsiveness of its other receptors accordingly. The signaling pathways via which NKG2D mediates its effects on other receptors are only beginning to be unraveled, especially in NK cells. Future studies must reveal how we will be able to exploit NKG2D-based therapies, without introducing unforeseen effects.

## Author Contributions

VJ wrote the section on NK cells. FW wrote the section on T cells. BP wrote the other sections and edited the article.

## Conflict of Interest Statement

The authors declare that the research was conducted in the absence of any commercial or financial relationships that could be construed as a potential conflict of interest.
